# Sublethal Effects of Hexaflumuron on Adults of *Apolygus lucorum* (Hemiptera: Miridae)

**DOI:** 10.3390/toxics13100846

**Published:** 2025-10-05

**Authors:** Xie Wang, Yu Gao, Yongqiang Liu, Peiling Wang, Yanhui Lu

**Affiliations:** 1 College of Agriculture, Shihezi University, Shihezi 832003, China; 2 State Key Laboratory for Biology of Plant Diseases and Insect Pests, Institute of Plant Protection, Chinese Academy of Agricultural Sciences, Beijing 100193, China

**Keywords:** insect growth regulators, sublethal effects, acute toxicity, longevity, fecundity

## Abstract

Hexaflumuron, a benzoylurea insecticide, exhibits high toxicity against various insect pests. Sublethal doses of hexaflumuron significantly impair nymphal development and subsequent fecundity in the resulting *Apolygus lucorum* (Meyer-Dür) adults. However, the effects on adults of *A. lucorum* that are exposed to sublethal concentrations of hexaflumuron are not known. In this study, we evaluated the toxicity of hexaflumuron to 1st- and 3rd-instar nymphs, as well as one-day-old adults, of *A. lucorum* using an artificial diet with hexaflumuron incorporated. Acute toxicity bioassays determined the median lethal concentration (LC_50_) for 1st- and 3rd-instar nymphs of *A. lucorum* to be 0.311 and 0.337 mg/L. In contrast, the LC_50_ level for one-day-old adults of *A. lucorum* was 13.193 mg/L. To evaluate the sublethal effects of hexaflumuron on 3rd-instar nymphs, we used LC_10_, LC_20_, and LC_50_ concentrations (0.018, 0.127, and 0.337 mg/L), fed for 48 h. The exposure of 3rd-instar nymphs to the LC_50_ concentration significantly reduced the fecundity of adults. We also exposed one-day-old adults to LC_10_, LC_20_, and LC_50_ dietary concentrations (0.089, 4.587, and 13.193 mg/L) for 48 h. Exposure to the LC_10_ and LC_20_ concentrations caused significant reductions in longevity and fecundity in adult females. The LC_50_ concentration also caused a significant reduction in the longevity of adult males and the egg hatching rate. An understanding of these sublethal impacts can facilitate the development of effective control strategies targeting *A. lucorum*.

## 1. Introduction

*Apolygus lucorum* (Meyer-Dür) (Hemiptera: Miridae) is a polyphagous, sap-feeding herbivore that causes significant damage in several crops [1]. It feeds on over 200 plant species, including cotton, fruit trees, and vegetable crops [2]. Both adults and nymphs of *A. lucorum* feed on the meristems of plant terminals, as well as young cotton buds, immature fruits, and other tissues. Their feeding results in bushy plants and the abscission of cotton buds and young fruits [3]. Since the commercial release of Bt cotton (expressing Cry toxins targeting lepidopteran pests such as *Helicoverpa armigera* (Hübner) in 1997, the pest status of mirid bugs has changed from minor cotton pests to dominant pest species in the Yangtze and Yellow River basins in China [4]. At present, *A. lucorum* is managed exclusively using insecticides, leading to the development of pesticide resistance and environmental contamination. Therefore, it is imperative that we explore novel environmentally friendly insecticides and their sublethal effects, as they are of considerable economic and ecological importance in establishing sustainable integrated control strategies for *A. lucorum*.

Compared to many conventional insecticides, insect growth regulators (IGRs) have a more specific mode of action against pests and are not highly toxic to non-target organisms [5]. IGRs interrupt molting (functioning as juvenile hormone or ecdysone mimics) and disrupt cuticle formation (as chitin synthesis inhibitors) or affect insect endocrine systems [6]. One such IGR is hexaflumuron, which is a benzoylphenylurea derivative that interferes with chitin synthesis during molting, eventually causing the death of affected insects [5,7,8]. In recent years, the effectiveness of hexaflumuron against many insect pests has been widely recognized [9,10,11]. The sublethal effects of hexaflumuron have also been examined, including in some species of Lepidoptera, Isoptera, and Orthoptera. For instance, Mahmoudvand et al. [12] demonstrated that the sublethal effects of hexaflumuron on the diamondback moth (*Plutella xylostella* L.) (Lepidoptera: Yponomeutidae) included prolonged developmental duration and reduced longevity. Bakr et al. [13] showed that nymphs of *Schistocerca gregaria* Forskål (Orthoptera: Acrididae) exhibited prolonged nymphal durations when treated with hexaflumuron, and none successfully reached the adult stage.

Given their mode of action, only insects in immature stages are affected by IGRs, which do not directly kill adults. Mirid adults are highly mobile, have high longevity and a broad host range, and inflict cryptic damage that is not immediately visible [14]. Given these traits, adult mirids may frequently be exposed to low concentrations of insecticides in the field, which may have sublethal effects [15]. According to Tan et al. [5], exposure to sublethal hexaflumuron concentrations resulted in a significant prolongation of nymphal development and lower weights in 5th-instar *A. lucorum* nymphs. In addition, Dang et al. [16] found that 2nd-instars of *A. lucorum* treated with low doses of hexaflumuron exhibited slower developmental rates and the subsequent adults were less fecund and showed shorter longevity. Survival was also reduced in treated 4th-instar nymphs. Catchot et al. [17] found that the exposure of adults of the tarnished plant bug (*Lygus lineolaris* Palisot) to the IGR novaluron reduced egg viability regardless of adult age or exposure route. However, whether hexaflumuron induces sublethal effects in adults of *A. lucorum* remains unknown. In this study, we measured the direct toxicity of hexaflumuron to 1st- and 3rd-instars, as well as to one-day-old adults, of *A. lucorum* using artificial diets amended with labeled rates of hexaflumuron. Additionally, we investigated the sublethal effects of lower concentrations of hexaflumuron on 3rd-instar nymphs and one-day-old adults. Our study provides a theoretical basis for the field application of hexaflumuron for the control of *A. lucorum* populations.

## 2. Materials and Methods

### 2.1. Insect Rearing and Insecticide Preparation

Adults or nymphs of *A. lucorum* were collected from cotton fields in Langfang Experimental Station, Institute of Plant Protection, Chinese Academy of Agricultural Sciences (CAAS; 39.53° N, 116.70° E) (Langfang City, Hebei Province, China), to initiate our laboratory colony. Mirids was reared on the green bean (*Phaseolus vulgaris* L.) cultivar ‘Jia Dou Wang’ at 25 ± 1 °C, with 60 ± 5% RH and a 14:10 h (L:D) photoperiod. Hexaflumuron (98% pure) was purchased from Hebei Veyong Biochemical Co., Ltd. (Shijiazhuang, China).

### 2.2. Acute Toxicity of Hexaflumuron to A. lucorum

The acute toxicity of hexaflumuron to 1st-instar nymphs, 3rd-instar nymphs, and one-day-old *A. lucorum* adults was assessed using an artificial diet to which hexaflumuron had been added. First, a hexaflumuron stock solution (10 g/L) was prepared by dissolving hexaflumuron in acetone. As indicated in our preliminary experiments, adults of *A*. *lucorum* were significantly less susceptible to hexaflumuron than nymphs. For tests with 1st- and 3rd-instars, hexaflumuron was diluted with a liquid artificial diet, forming a series of concentrations: 0.1, 0.2, 0.4, 0.6, and 0.8 mg/L. For one-day-old adults, the tested concentrations were higher: 3.125, 6.25, 12.5, 25, and 50 mg/L. The artificial diet without hexaflumuron was used as the control (CK). These diets were formulated in Parafilm packets (Pechiney Plastic Packaging, Menasha, WI, USA) that were placed inside transparent glass containers (3 cm dia, 5 cm height); each container held one diet packet. After 5 h of starvation, three *A. lucorum* nymphs or adults were transferred into these containers. Each treatment (i.e., concentration × life stage) was repeated three times. Each replicate contained 10 test dishes (i.e., a container with a diet packet and three individual insects) (1st- or 3rd-instar nymphs or adults), meaning a total number of 90 test insects of a given life stage per treatment. All test containers were maintained in a climate-controlled chamber at 25 ± 1 °C, with 60 ± 5% RH and a 14:10 (L:D) h photoperiod. After 48 h, we replaced the diet packets with fresh green beans (2 cm sections cut with a scalpel) for post-exposure rearing. Mortality was recorded at 24, 48, 72, and 96 h. Nymphs or adults that did not move when touched with a thin brush were regarded as dead.

### 2.3. Sublethal Effects of Hexaflumuron on Adults That Developed from 3rd-Instar Nymphs of A. lucorum

The sublethal effects of three concentrations of hexaflumuron—LC_10_ (0.018 mg/L), LC_20_ (0.127 mg/L), and LC_50_ (0.337 mg/L)—were evaluated for adults that developed from nymphs that survived pesticide exposure as 3rd-instars. We achieved this by treating 3rd-instar nymphs of *A. lucorum* and then testing the adults that developed from the surviving nymphs. These concentrations were prepared as described above in relation to testing higher doses for acute toxicity. The liquid artificial diet without hexaflumuron was used as the control (CK).

Each treatment (the three sublethal concentrations) was replicated four times, and there were 20 pairs of adults (from treated nymphs) for each replicate, meaning a total number of 80 pairs of adults for each treatment. For tests, we placed 3rd-instar nymphs in transparent glass containers (3 cm dia, 5 cm height), each of which included one diet packet of the desired concentration. After 48 h, the survivors of each treatment were individually transferred into smaller glass containers (2.5 cm dia, 7.5 cm height) in which one 2 cm section of a green bean free from insecticide residues was present and then held for observation. After adults had emerged from the treated nymphs, they were paired (1 male and 1 female) in new transparent glass containers (3 cm dia, 5 cm height). Adults were supplied daily with fresh green beans as food and an oviposition site. The eggs found in green beans after 24 h exposure to adults were checked daily and counted under a microscope. This process was repeated until all parental females had died. Eggs in beans were housed in new glass containers (2.5 cm dia, 7.5 cm height), and after all viable eggs had hatched, the number of nymphs divided by the number of eggs found was taken as the egg hatching rate. The experiment was conducted in a climate-controlled chamber at 25 ± 1 C, with 60 ± 5% RH and a 14:10 (L:D) h photoperiod.

### 2.4. Sublethal Effects of Hexaflumuron on One-Day-Old Adults of A. lucorum

To assess sublethal effects on directly treated adults, we tested three concentrations—LC_10_ (0.089 mg/L), LC_20_ (4.587 mg/L), and LC_50_ (13.193 mg/L)—using one-day-old adults of *A. lucorum*. These concentrations were prepared following the method described in the section on testing acute toxicity. The artificial diet without hexaflumuron was used as the control (CK). We placed one-day-old adults into transparent glass containers (3 cm dia, 5 cm height), each of which included one diet packet. After 48 h, the survivors of each treatment were paired (1 male and 1 female) in new transparent glass containers (3 cm dia, 5 cm height) with one 2 cm section of green bean for rearing. Thereafter, the number of eggs laid by individual females was recorded, as well as the number of eggs that successfully hatched into nymphs, until all females had died. The experiment was conducted in a climate-controlled chamber at 25 ± 1 °C, with 60 ± 5% RH and a 14:10 (L: D) h photoperiod. Each treatment was replicated four times, with 20 pairs of adults in each replicate, meaning a total of 80 pairs of adults for each treatment.

### 2.5. Statistical Analysis

Data obtained from the acute toxicity test allowed us to calculate lethal concentrations (LC_10_, LC_20_, LC_50_, LC_90_) for 1st- and 3rd-nymphal instars and one-day-old adults, including the toxicity regression line, 95% confidence limits, Chi-squared values (*χ*^2^), and degrees of freedom (*df*) based on mortality data across concentration treatments using prohibit analysis with SPSS 26.0 software. The data for female or male longevity (days from adult eclosion to death), fecundity (the total number of eggs laid during the female lifespan), and egg hatching rate (the number of emerged 1st-instar nymphs divided by the total number of eggs) for *A. lucorum* followed a normal distribution, and one-way analysis of variance (ANOVA) was applied, followed by Tukey’s HSD to compare treatment means. ANOVA was carried out using R 4.2.2 software.

## 3. Results

### 3.1. Acute Toxicity

**Figure 1:**
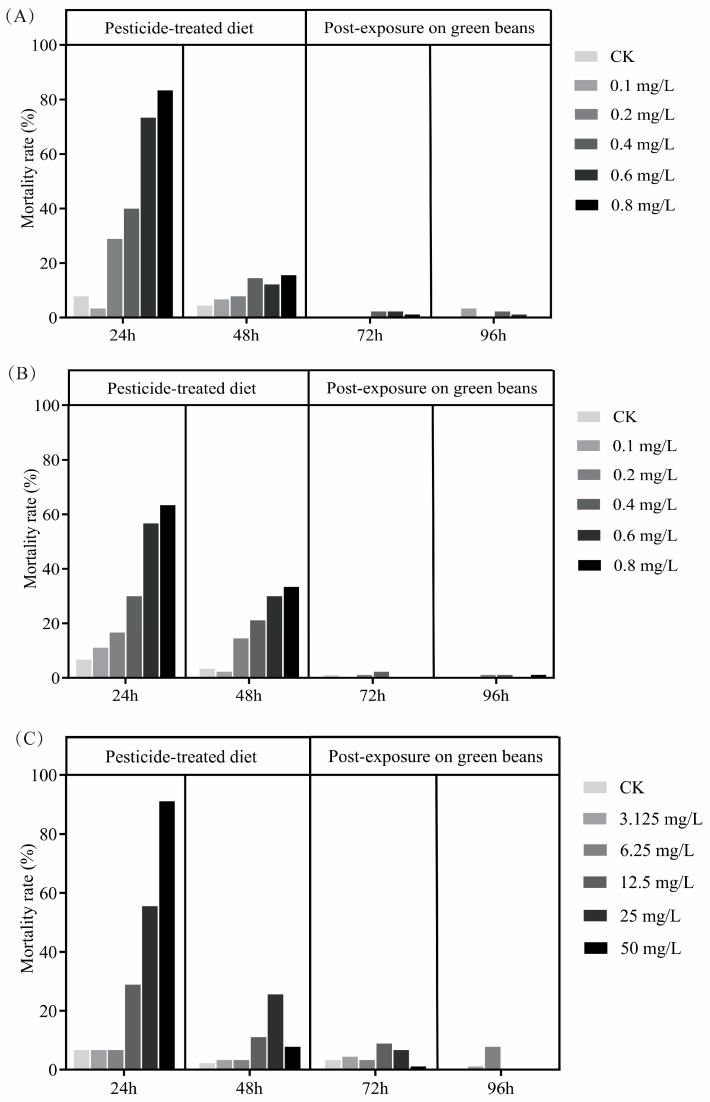
Acute toxicity rates for *A. lucorum* life stages treated with various concentrations of hexaflumuron in a liquid diet for 48 h, followed by further post-exposure observation of mortality over an additional 48 h (96 h from the start of experiment, with the last 48 h being on green beans). (**A**) 1st-instar nymphs, (**B**) 3rd-instar nymphs, (**C**) one-day-old adults.

Following continuous dietary pesticide exposure for the first 48 h (and then 48 additional hours of post-exposure observation while feeding on a clean diet), the mortality of 1st- and 3rd-instar nymphs and one-day-old adults of *A. lucorum* increased progressively with rising concentration. However, nearly all mortality was clustered in the first 48 h. After 24 h of feeding on a pesticide-contaminated diet, mortality exceeded 60% for both nymphs and adults. After 48 h of feeding on that diet, mortality under the highest concentration of hexaflumuron reached over 90% for all three life stages (Figure 1).

For 1st-instars, probit analysis of mortality rates after feeding for 48 h on a pesticide-contaminated liquid diet found LC_10_, LC_20_, LC_50_, and LC_90_ values of 0.013, 0.116, 0.311, and 0.609 mg/L, respectively. For 3rd-instars, the LC_10_, LC_20_, LC_50_, and LC_90_ values were 0.018, 0.127, 0.337, and 0.655 mg/L, respectively. For one-day-old adults, the above values were 0.089, 4.587, 13.193, and 26.902 mg/L, respectively (Table 1).

### 3.2. Sublethal Effects of Hexaflumuron on Adults That Developed from Nymphs That Survived Pesticide Exposure as 3rd Instars

**Figure 2:**
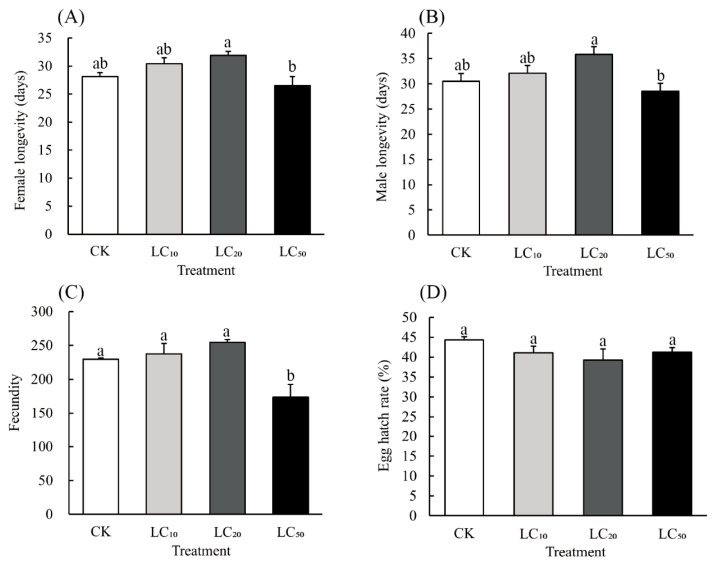
Effects of hexaflumuron treatment in adults that survived exposure as 3rd-instar nymphs to three *A. lucorum* concentrations: LC_10_ (0.018 mg/L), LC_20_ (0.127 mg/L), and LC_50_ (0.337 mg/L): (**A**) female longevity, (**B**) male longevity, (**C**) fecundity (egg number/female), and (**D**) egg hatching rate. Eighty pairs of adults were assessed for each pesticide concentration. Data are presented as means ± SE; different lowercase letters above whiskers indicate significant differences (*p* < 0.05, Tukey’s HSD).

Following 48 h of hexaflumuron exposure as 3rd-instar nymphs, surviving adults, in some cases, showed significant variation in adult longevity among hexaflumuron concentrations. This was seen in both females (F_3,12_ = 5.332, *p* = 0.015) and males (F_3,12_ = 4.152, *p* = 0.031). Similarly, differences were observed in the fecundity of females of this insect treated in this manner among the different hexaflumuron concentrations (F_3,12_ = 7.893, *p* = 0.004). Compared to the control (CK), no difference was detected in longevity for any hexaflumuron concentration (Figure 2A,B). The fecundity of females exposed as 3rd-instars to the LC_50_ rate of hexaflumuron did differ significantly from the control; it was reduced by 30.4% (Figure 2C). There were no significant differences among the treatments in egg hatching rates (F_3,12_ = 1.438, *p* = 0.280) (Figure 2D).

### 3.3. Sublethal Effects of Hexaflumuron on Adults That Survived Pesticide Exposure When Treated as One-Day-Old Adults

**Figure 3:**
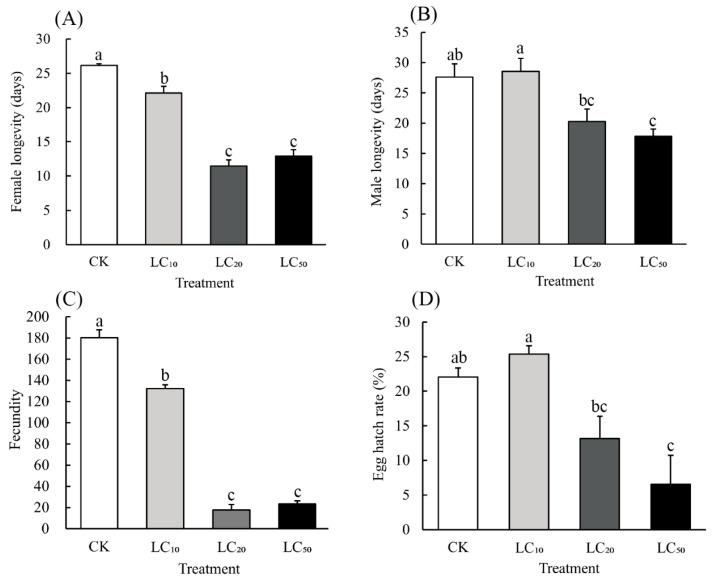
Effects of hexaflumuron treatment at varying concentrations after treatment of one-day-old adults of *A. lucorum*: (**A**) female longevity; (**B**) male longevity; (**C**) fecundity (eggs/female); and (**D**) egg hatching rate. This experiment was conducted using hexaflumuron at LC_10_ (0.089 mg/L), LC_20_ (4.587 mg/L), and LC_50_ (13.193 mg/L) concentrations against one-day-old adults of *A. lucorum*. Eighty pairs of insects were used for each treatment in the experiment. Data are presented as means ± SE; different lowercase letters above whiskers indicate significant differences (*p* < 0.05, Tukey’s HSD).

For *A. lucorum* individuals treated as one-day-old adults, the longevity of both females (F_3,12_ = 77.301, *p* < 0.001) and males (F_3,12_ = 7.557, *p* = 0.004), the fecundity of females (F_3,12_ = 250.985, *p* < 0.001), and the egg hatching rate (F_3,12_ = 9.454, *p* = 0.002) all differed significantly among the hexaflumuron concentrations. The female longevity after LC_10_, LC_20_, and LC_50_ treatments was 15.3%, 56.2%, and 50.7% lower than the control (Figure 3A), respectively, whereas the male longevity after the LC_20_ and LC_50_ treatments was 26.7% and 35.4% lower than the control (Figure 3B). Compared with the control, the fecundity of treated one-day-old adults decreased by 26.6%, 90.3%, and 87.1% (Figure 3C) under LC_10_, LC_20_, and LC_50_ treatments, respectively, while both LC_20_ and LC_50_ treatments reduced the egg hatching rate by 40.4% and 70.3% (Figure 3D).

## 4. Discussion

Following pesticide application, the uneven distribution and continuous degradation of active ingredients on plants often result in herbivore populations being exposed to low and/or sublethal pesticide concentrations [15,18]. These residues cause sublethal effects that may manifest through either the negative suppression of pests or positive stimulation of development and reproduction in insect populations [19]. As an insect growth regulator (IGR), hexaflumuron has been widely used to control various agricultural pests [11,20,21]. Previous studies have demonstrated that sublethal doses of hexaflumuron significantly impair the growth and development of *A. lucorum* nymphs [5,16]. In our study, exposure of one-day-old adults of *A. lucorum* to sublethal concentrations of hexaflumuron resulted in significant reductions in longevity (in both females and males), fecundity, and egg hatching rate (Figure 3). This finding is similar to that of Catchot et al., who observed that sublethal concentrations of novaluron (a type of IGR) reduced both the fecundity and egg hatching rate of newly emerged adults of the tarnished plant bug [18]. Furthermore, in another laboratory study [22], they showed that the ingestion of novaluron reduced potential oviposition by newly emerged females by reducing chitin synthesis in the ovarian tissue and thereby disrupting oocyte development. In general, our results illustrate that hexaflumuron effectively impacted the development and reproduction of *A. lucorum*, both when they were treated directly and when they were treated as 3rd-instars and then studied once survivors reached adulthood.

The LC_50_ values for direct toxicity to 1st-instars, 3rd-instars, and one-day-old adults were 0.311, 0.337, and 13.193 mg/L, respectively (Table 1), indicating that early instars were more susceptible to such effects of hexaflumuron, likely due to their smaller body size. As an IGR, hexaflumuron disrupts chitin formation in the insect cuticle, effectively inhibiting the molting process and consequently exhibiting greater toxicity against early instars [11], which must molt. Notably, our findings on sublethal effects indicated a non-significant tendency toward prolonged longevity in both female and male adults (that had emerged from 3rd-instar nymphs exposed to hexaflumuron), as well as fecundity, when insects were treated with LC_10_ and LC_20_ concentrations. This finding may be attributable to the stimulatory effects (hormesis) induced by low doses of hexaflumuron in *A. lucorum* [23]. Hormesis refers to the possible stimulation of organism performance occurring at low levels of exposure to toxic agents that are normally harmful at high levels of exposure [24]. However, no such trend was observed in the egg hatching rate. This lack of effect may be attributed to the mode of action of hexaflumuron as a chitin synthesis inhibitor that disrupts physiological processes such as oogenesis, ultimately preventing the insects from reaching reproductive maturity.

Given their long lifespan, dispersal capabilities, and damage potential, *A. lucorum* adults constitute prime targets for area-wide integrated pest management (IPM) [2]. The population dynamics of *A. lucorum* are primarily determined by the diversity and flowering phenology of locally available host plants. Throughout their life cycle, individual *A. lucorum* mirids undergo extensive host plant switching, with adults tracking a succession of flowering plant species within the agricultural landscape [25,26]. Based on these findings, it can be inferred that if adults of *A. lucorum* are exposed to sublethal concentrations of hexaflumuron on one of more of these host plants, they will potentially suffer reduced female longevity and lower fecundity. These adults, as they migrate among crop fields, suffer an accumulation of such negative effects if fields are treated with IGRs, ultimately leading to a decline in the species’ population density. These effects may mean that the widespread use of hexaflumuron could play an important role in the regional management of *A. lucorum*.

Cotton fields in China host a diverse complex of mirid bugs, primarily including *A. lucorum*, *Adelphocoris suturalis* Jakovlev, *Adelphocoris lineolatus* Goeze, *Adelphocoris fasciaticollis* Reuter, and *Lygus pratensis* L. [14]. Given that previous studies have proven hexaflumuron’s consistent insecticidal efficacy against these mirid bug species [27], it is likely that its widespread use would also suppress populations of other mirid bug species in cotton fields. While our study has clarified the sublethal effects of hexaflumuron on *A. lucorum*, its primary limitation is that all experiments were conducted in laboratory conditions, lacking validation based on field trials. Complex factors in the field, such as fluctuations in temperature and humidity, rainfall, the activity of natural enemies, and the crop ecosystem, may all influence the ultimate efficacy of this pesticide. Therefore, caution is warranted when extrapolating these laboratory findings to practical field applications.

## 5. Conclusions

This study demonstrates that hexaflumuron induces sublethal effects in adults of *A. lucorum*, leading to reduced female longevity and decreased fecundity. These findings provide a theoretical basis for the field application of hexaflumuron in controlling populations of *A. lucorum* and potentially other mirid species.

## Figures and Tables

**Table 1:** Acute toxicity (mg/L) of hexaflumuron to *A. lucorum* treated as 1st- or 3rd-instar nymphs or as one-day-old adults, at 48 h post exposure.

	LC Values (mg/L) and 95% CL					
Life Stage	Slope ± SE	LC_10_	LC_20_	LC_50_	LC_90_	*χ* ^2^
1st-instars	4.303 ± 1.339	0.013	0.116	0.311	0.609	6.655
		(N/A~0.058)	(0.073~0.152)	(0.281~0.343)	(0.560~0.671)	
3rd-instars	4.023 ± 1.354	0.018	0.127	0.337	0.655	3.777
		(N/A~0.065)	(0.082~0.165)	(0.304~0.370)	(0.603~0.721)	
One-day-old adults	0.098 ± 1.290	0.089	4.587	13.193	26.902	1.387
		(N/A~1.969)	(2.799~6.085)	(11.762~14.836)	(23.560~30.012)	

Ninety insects per treatment were used in the experiment. N/A: The lower limit of the confidence interval is biologically meaningless and is therefore not reported.

## Data Availability

The data presented in this study are available on request from the corresponding author.
